# Proprioceptive Movement Illusions Due to Prolonged Stimulation: Reversals and Aftereffects

**DOI:** 10.1371/journal.pone.0001037

**Published:** 2007-10-17

**Authors:** Tatjana Seizova-Cajic, Janette L. Smith, Janet L. Taylor, Simon C. Gandevia

**Affiliations:** 1 Prince of Wales Medical Research Institute and University of New South Wales, Sydney, New South Wales, Australia; 2 School of Psychology, University of Sydney, Sydney, New South Wales, Australia; Harvard Medical School, United States of America

## Abstract

**Background:**

Adaptation to constant stimulation has often been used to investigate the mechanisms of perceptual coding, but the adaptive processes within the proprioceptive channels that encode body movement have not been well described. We investigated them using vibration as a stimulus because vibration of muscle tendons results in a powerful illusion of movement.

**Methodology/Principal Findings:**

We applied sustained 90 Hz vibratory stimulation to biceps brachii, an elbow flexor and induced the expected illusion of elbow extension (in 12 participants). There was clear evidence of adaptation to the movement signal both during the 6-min long vibration and on its cessation. During vibration, the strong initial illusion of extension waxed and waned, with diminishing duration of periods of illusory movement and occasional reversals in the direction of the illusion. After vibration there was an aftereffect in which the stationary elbow seemed to move into flexion. Muscle activity shows no consistent relationship with the variations in perceived movement.

**Conclusion:**

We interpret the observed effects as adaptive changes in the central mechanisms that code movement in direction-selective opponent channels.

## Introduction

The level of adaptation of a sensory system depends on the statistics of past stimulation-its sensory ‘diet’-and it can be defined operationally in terms of a stimulus evoking a neutral or indifferent response [1]. A well-known example comes from vision: after adaptation to a stimulus moving continuously in one direction, a subsequent stationary stimulus appears to move in the opposite direction [2]. We characterize a similar phenomenon for perception of limb movement in the domain of proprioception.

Adaptation in the proprioceptive system has been extensively studied for *active* movements [for review see 3], and to a lesser degree, for perception of position [e.g., 4] and postural control [e.g., 5]. Proprioceptive adaptation in conscious perception of movement with muscles relaxed and thus free from an efferent contribution from the motor system has been little explored.

A widely-used tool for investigation of movement perception is tendon vibration; it activates muscle spindle endings and induces an illusory sensation of movement [6]–[9]. Vibration lasting 30 seconds results in a decreased firing rate of muscle spindle primaries lasting for about 40 seconds [10]. This peripheral adaptation is likely to have a perceptual counterpart [11]; furthermore, it is likely that adaptation also occurs at the supraspinal levels. Earlier research offered only brief qualitative reports to suggest that perceptual adaptation occurs, such as variations in the sensation of movement during vibration [7]–[9] and a transient perception of movement in the opposite direction after vibration [6]–[8]. For example, Goodwin and colleagues [6], who gave a thorough description of vibration-induced movement illusions, reported that “after the end of a period of vibration there is often a sensation lasting a second or so that the arm had reversed its direction of motion … but the illusion was too transitory for us to make any effective observations upon it.” (p. 725).

Our aim was to fill the gap in knowledge about adaptation to movement stimuli in the proprioceptive system. We used quantitative methods to investigate adaptation to a long-lasting movement signal induced by vibration. Adaptation has been successfully used as a tool to infer the properties of visual cortical processes in humans [12], which are at this stage difficult to investigate using neurophysiological methods. Our detailed description of adaptation phenomena in proprioception can be used in a similar way to expose the likely central processing.

A recent study by Kito and colleagues [13], of which we were not aware at the time when we conducted ours, was based on a similar rationale. This group conducted a psychophysical and transcranial magnetic stimulation study of the aftereffect following hand movement induced by tendon vibration. They confirmed the existence of the aftereffect but found that, rather than ‘a second or so’, its average duration was up to 5 seconds, depending on the duration of the preceding vibration (up to 60 seconds). Stimulation of the motor cortex showed that responses in the non-vibrated antagonist of the vibrated muscle increased during vibration and decreased thereafter. The imbalance in excitability between the two muscles correlated with the illusory movements. The authors concluded that an imbalance in the cortical processing of spindle input was responsible for the aftereffect and compared this explanation with the ‘fatigue model’ of the visual aftereffects (p. 82).

Our study is primarily psychophysical and it investigates adaptation *during* a long-lasting stimulation as well as the aftereffect. We applied vibration for a long period of time (6 minutes) and recorded modulations in perceived movement throughout this period as well as after vibration, with some surprising results suggesting further analogies between processing of motion in vision and proprioception. Specifically, we found that *during* vibration, ‘reversals’ of movement were occasionally perceived. Thus the unchanging stimulation resulted in a changing percept. In a follow-up experiment we also recorded electromyographic activity (EMG) from biceps and triceps to determine whether it correlated with perceptual effects.

## Results

Participants reported by keypress the direction and speed of illusory arm movement, if any, throughout a 6-minute period of vibration of the biceps tendon, and in the 2 minutes after the vibration. They used one key to indicate elbow extension, and the other to indicate flexion. The frequency of keypresses indicated the relative speed of the perceived movement. In the absence of a movement sensation, keypresses stopped.

**Figure 1:**
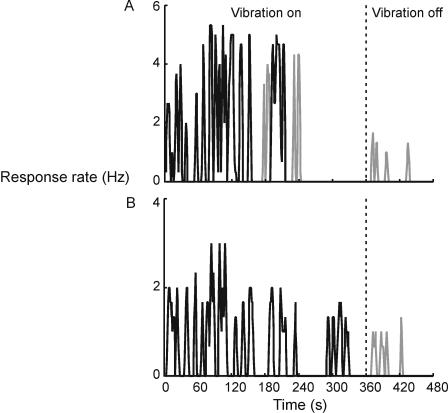
Sample of individual results. Raw rates of keypress, recorded with 1 second precision, have been smoothed using a 3-second window. Black line indicates perceived movement to the left (extension) and the grey line, movement to the right (flexion). The vibration was switched off after 360 seconds and the participants were required to respond for a further 120 seconds. A. Results from Run 1. B. Results from Run 2.

Vibration of the biceps induced the expected illusion of elbow extension. Representative individual results shown in **Figure 1** indicate that the illusion (black line) waxed and waned during the 6-minute vibration period, with periods of no movement occurring more often with prolonged stimulation. The perceived speed of movement varied but did not change progressively in consecutive bursts of movement. Surprisingly, there were occasional reversals in the direction of illusory motion during vibration (grey line in the ‘Vibration on’ period). Cessation of vibration was followed by a movement aftereffect (grey line in the ‘Vibration off’ period). Group data presented below confirm and extend these observations.

### Vibration induces illusions of movement

Two kinds of responses occurred *during* vibration, extensions and flexions (reversals). In addition, a lack of response for periods of three seconds or longer indicated that no movement was perceived. On average, the illusion of extension was present for 48% (±25% SD) of total vibration time in Run 1 and illusion of flexion for 9% (±10% SD) of the time. The corresponding values in Run 2 are 42% (±24%) and 4% (±9%).

### Frequency and duration of illusory movements changes with prolonged stimulation

**Figure 2:**
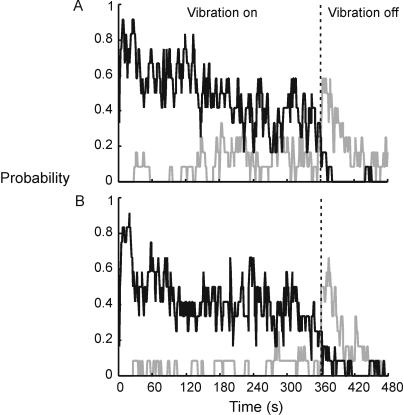
Probability of responding as a function of time recorded with 1-second precision (N = 12). Black line indicates probability of perceived elbow extension (Pext) and the grey line, of flexion (Pflex). Participants had the option of not pressing either key (‘silence’), in the absence of a clear feeling of movement. Thus, for any given second, Pext+Pflex+Psilence = 1. A. Results from Run 1. B. Results from Run 2.

The probability of occurrence of illusory movement among the participants is shown in **Figure 2**. Vibration was most likely to induce an illusion of arm movement in the expected direction (extension) in the first 30–40 s. From the initial value of ∼0.8, the probability of experiencing extension gradually declined to ∼0.4 at the end of the 6-minute vibration period. This decline was compensated by the opposite trend in the periods of no movement and reversals.

The changing pattern of responses with ongoing vibration is shown in **Table 1**. Extension responses were initially by far the greatest in number and duration. Within 30 s of the start of vibration in Run 1 the participants experienced the onset of a total of 19 bursts of illusory extension lasting a total of 472 s (note that the onset, but not necessarily the end of these events fell within the 30 s). Extension responses decreased with time and this was compensated for by the opposite trend in the silent periods and flexion responses. The first reversal experienced by each subject occurred after a relatively long delay (median  = 123 s; range 28–215 s), and the number of participants experiencing reversals increased with time (see Figure 2). The reversals usually occurred after periods in which no movement was perceived.

**Table 1:** Frequency and duration of movement illusions and silent periods

	Extension		Flexion		No movement	
	n	Total duration (sec)	n	Total duration (sec)	n	Total duration (sec)
Onset between 1–30 sec	19	472	2	28	14	84
Onset between 120–150 sec	19	146	6	42	22	175
Onset between 240–270 sec	14	147	4	32	15	223

Frequency and total duration of movement illusions and silent periods in Run 1 is shown as a function of the time of the onset of the event. ‘Onset’ indicates the time of onset of the event relative to the moment when vibration started (the whole duration of the event was measured, regardless of whether it finished within the 30 sec period or not). Note that extension responses initially dominated but were later replaced by the increasingly longer ‘no movement’ periods and, to a lesser extent, flexion responses.

Comparison between the runs reveals that *any* illusory movement was less likely to be experienced in Run 2. The mean probability of perceived elbow extension was lower in Run 2: 0.43 compared to 0.50 in Run 1 (t(359) = 8.22; p<0.001). The corresponding values for reversals were 0.04 and 0.10 (t(359) = 12.38, p<0.001).

**Figure 3:**
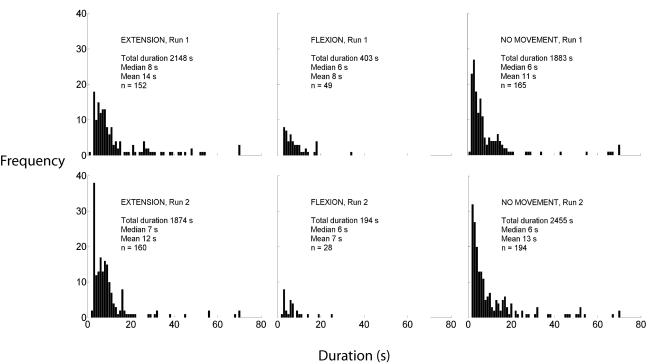
Histograms showing durations of individual instances of Extension, Flexion and No movement periods during adaptation. The upper limit for the x-axes has been set at 80 because there were only 9 points above that value (either Extensions or No movement occurrences).

Individual bursts of movement tended to be shorter in Run 2 (see **Figure 3** **)**. Their duration in both runs was usually less than 10 s, with group median values of 6–8 s, and a long tail of events of greater duration. Periods of no movement followed a similar, positively skewed distribution. The difference between the mean duration of the extension responses in the two runs was significant at the 0.05 level (calculated by the bootstrap method based on 1000 samples), while the differences for flexion responses and no-movement periods were not significant.

### Illusory movement occurs in bursts of undiminished velocity during vibration

**Figure 4:**
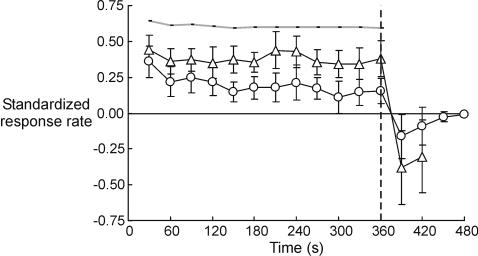
Mean response rate indicating the velocity of illusory movement, Run 1 and Run 2 combined data (N = 12). Data points represent standardized mean rate of keypress for the preceding 30-seconds period. Error bars represent 95% confidence intervals of the mean. We only show the results from time periods with a probability of responding of more than 0.5 across the two runs. *Circles*: Means were computed from the responses indicating illusory movement to the left (positive sign) and the right (negative sign), as well as no movement (zeros). The velocity computed this way steadily decreases throughout the vibration period. *Triangles*: Means were computed only for periods in which movement was perceived (i.e., zeros were excluded) separately for illusory extension (positive sign) and flexion (negative sign). Perceived speed measured this way remains relatively constant. *Thick grey line:* results from the control condition in which the participants attempted to press one key at a constant rate for six minutes.

Given that the frequency of illusory extensions decreases over time, it is not surprising that an overall perceived velocity, computed taking into account no-movement periods (see Methods section), decreases. **Figure 4** (circles) shows that the mean velocity for the combined data from the two runs steadily decreases throughout the vibration period (the first 360 s), following a significant linear trend (F(1,11) = 14.15, p<0.01). However, perceived speed measured only during ‘bursts’ of movement, i.e., when periods of no movement are excluded, remained relatively constant (see Figure 4, triangles; responses with a probability smaller than 0.5 are not shown). We found no evidence of its linear increase or decrease with time (F(1,11) = 0.32).

There was also no linear trend in the control condition (thick grey line in Figure 4; F(1,11) = 0.32). On average, when requested, the participants could maintain a relatively constant rate of keypress for 6 min.

### Movement illusion is followed by an aftereffect

After vibration, about 60% of participants experienced an illusion of arm movement in the opposite direction (flexion) in Run 1. After ∼20 s, this aftereffect decreased sharply (Figure 2). A similar pattern occurred in the second run, but over the two minutes after vibration, the mean probability of experiencing the aftereffect was lower (0.20 compared to 0.23 in the first run; t(119) = 2.96, p<0.005).

In the post-vibration period, the illusion of flexion was present on average for 22% (±0.24% SD) of total time, and illusion of extension for only 2% (±0.06%) of the time. Individuals with longer periods of perceived extension *during* vibration also experienced longer periods of the flexion *after* vibration with a correlation of 0.72 (p<0.01).

The perceived speed of the movement aftereffect during the first 30 s after vibration is similar to that of the movement experienced during vibration (Figure 4). Figure 4 also shows that the aftereffect reaches its peak subjective velocity within 30 s after vibration, decreasing thereafter.

### Muscle activity shows no consistent relationship with perceptual experience

**Figure 5:**
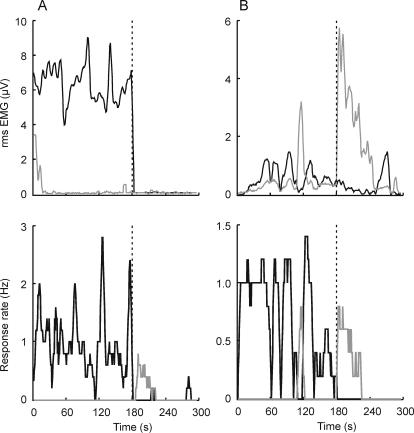
EMG records (top) and perceptual experience (bottom), individual data of two participants. Black line represents activity in the triceps and illusory extension, and grey line, biceps activity and illusory flexion. Data on the left (Participant 6 from Table 2) show positive correlation between biceps activity and the illusory extension (r = 0.60) and no other associations. Data on the right (Participant 1) show correlation between activity in the triceps and illusory extension (r = 0.51), as well as between the biceps and illusory flexion (r = 0.90).

Results of the main experiment were replicated in a follow-up experiment (N = 7), in which vibration lasted 3 minutes, post-vibration period 2 minutes, and EMG was recorded from the biceps and triceps muscles. EMG from one or the other muscle correlated with perceptual experience in some subjects some of the time, but showed little or no correlation in others (see **Table 2**). There is no consistent pattern present across all individuals. **Figure 5** presents data of two participants: Participant 6 (left panels) showed activity in the biceps during extension and little else, and Participant 1 (right panels) showed activity in the triceps during perceived extension and in the biceps during flexion.

**Table 2:** Correlation coefficients between rms EMG and direction of perceived movement reported at the time

Participant	Triceps		BICEPS	
	Extension	Flexion	Extension	Flexion
1	0.51	−0.25	−0.50	0.90
2	0.25	0.25	0.52	0.36
3	0.38	−0.24	0.30	0.27
4	0.62	−0.49	0.62	−0.48
5	−0.05	0.62	0.08	0.74
6	0.12	−0.06	0.73	−0.40
7	0.83	0.34	−0.82	0.31
Means	0.14	0.02	0.13	0.24

Correlations were calculated for the whole duration of the trial (including both vibration and post-vibration period). Even though some participants [e.g. 1], [4], [5], [7] exhibit a relatively strong association between EMG and perceived movement, it is not always in the same direction, and others show little relationship.

## Discussion

Stimulation of muscle spindles by vibration is known to generate illusions of limb movement in the direction that would stretch the vibrated muscle. We found that prolonged, continuous activation of muscle spindle endings with vibration results in a changing percept. The illusion of elbow extension comes in bursts or waves separated by periods of no illusory movement. Sometimes there is an illusory movement in the opposite direction (flexion), especially after a relatively long period of stimulation. The duration of waves of illusory extension decreases with time, both during ongoing vibration, and in a subsequent application of the same stimulus. With ongoing stimulation, the opposite movement directions cancel out, gradually bringing the average velocity closer to zero. However, perceived speed of movement during individual waves does not decrease. Cessation of stimulation is followed by an aftereffect. This consists of illusory movement in the opposite direction with a similar speed to the vibration-induced movement. The aftereffect lasts longer in individuals who experience longer total periods of movement during adaptation.

The findings concerning the aftereffect of vibration corroborate those of Kito and colleagues [13], even though the psychophysical method they used was different in a potentially significant way. Their subjects replicated the *extents* of illusory movement and aftereffect after each trial. Thus rather than directly reflecting experience of movement, the responses reflected a memory of perceived displacement, or perhaps displacement inferred from perceived movement. This ambiguity is undesirable because it is known that movement and displacement illusions are not necessarily equivalent, and may not depend on the same underlying mechanisms [6], [14]. Thus it is uncertain whether the findings of Kito et al were similar to ours because their participants relied on perceived movement, or because position aftereffect is similar to the movement aftereffect.

Our findings regarding the modulation in perceived movement during vibration, including reversals of perceived movement direction, are new. Combined with the aftereffect, these findings shed light on the mechanisms for processing signals of movement in the proprioceptive system. Their significance can be better understood if compared to vision in which adaptation has been used to probe the mechanism of sensory coding.

### Analogies and differences between vision and proprioception

In vision, two aspects of motion adaptation have been studied that might have proprioceptive analogues: a gradual decrease in the perceived velocity of a constantly moving pattern [15], and apparent motion of a stationary stimulus following exposure to a moving stimulus known as the motion aftereffect [for review see 16]. The proprioceptive analogue of the motion aftereffect, which indicates a shift in the neutral point, is that after stimulation, stationary limbs seem to move. However, the other aspect of visual adaptation, reduction in perceived speed, does not have an exact analogue in proprioception. Although the participants were less and less likely to experience illusory arm extension with ongoing vibration, there was no consistent decrease in perceived speed in those periods when the illusion was present. In each ‘wave’ of illusory movement, the speed quickly increased to a peak and decreased back to zero, usually within a period of 10 s, in spite of the ongoing stimulation. Furthermore, the whole event–a burst of movement–was repeated a number of times. Nothing similar has been described in vision. Notwithstanding this difference, another prominent feature is common to both vision and proprioception: puzzling reversals in perceived direction of motion during ongoing stimulation. Both in vision and proprioception, reversals have a long latency until they start, and a variable but usually short duration. The reversals in vision have only been described for stimuli with a repetitive pattern, and their interpretation is controversial [17], [18]. That they also occur in proprioception may help to explain what causes them in vision; a parsimonious explanation should apply to both modalities. Reversals belong to a broad family of multistable perceptual states, or incompatible alternating percepts that occur without a change in the stimulus. Multistable states have been much studied in vision (e.g., reversible figures [19], binocular rivalry [20]) and are a valuable tool for unveiling the organizational principles of the visual system, because the trigger for a change from one percept to another comes from within. We applied the same logic to our analysis of proprioceptive reversals, attributing them to adaptive processes discussed below.

### Adaptation in the proprioceptive movement channels

Barlow and Hill [21]; [see also 22] proposed a simple ratio model of the motion aftereffect in vision. According to the model, a temporary imbalance in the channels responsible for opposite directions of motion causes the motion aftereffect. A similar model applied to proprioception could potentially explain most of the findings presented here: the modulation in perceived speed during stimulation, the aftereffect and the reversals. There is evidence that perception of movement is determined by the weighted input from different channels encoding direction [23], [24]. A balance of inputs from synergist and antagonist muscles corresponds to the null point on the perception continuum, i.e. the absence of motion [25]. Tendon vibration increases the activity in the relevant channel and movement in one direction is perceived, but the response decreases with prolonged stimulation. This can explain the modulation in speed during a single wave of movement, although this modulation occurs at a short time scale in comparison to vision [15]. Conceivably, the diminishing response in the stimulated channel may at times even fall *below* the spontaneous firing rate in the opponent, non-stimulated channels, which could generate reversals. Another shift in overall activity occurs when stimulation ceases, because the adapted channel fires less than the others; this would produce the movement aftereffect. Even though the latter effect (perception of movement when none occurs) hardly seems adaptive, it is brought about by mechanisms that are essential to ensure efficient everyday functioning of sensory systems. According to this functional view of adaptation [1], [26]–[28], its effect is to minimize the response to the commonest state of the environment (or the body), and to increase sensitivity to change. In the present context, the proprioceptive input from the arm has been markedly altered by a period of intensive stimulation and the system's response is a gradual shift towards zero in the average response pattern. However, it did not reach a stable state, possibly because the stimulus duration was not long enough.

There is now also evidence that unbalanced excitability in the opponent *motor* channels correlates with perception. Kito and colleagues [13] found that the motor potential evoked by transcranial magnetic stimulation delivered over the motor cortex increased in the non-vibrated antagonist of the vibrated wrist muscle. The increased motor evoked potential (MEP) during vibration was followed by a decreased MEP after vibration, during the aftereffect. At the same time, there was little change in the vibrated muscle. This imbalance in the relative excitability of the corticospinal pathways to the opposing wrist muscles correlated with illusory movements, such that the direction of felt movement was consistent with contraction of the more excitable muscle. Similarly, Gilhodes, Roll and Tardy-Garvet [29] found increased activity (measured with EMG) in the antagonist of the vibrated muscle (the antagonist vibration reflex, as opposed to the tonic vibration reflex recorded in the agonist). Although in the present study we also found a relationship between EMG and perception in some subjects, it was inconsistent across and even within subjects, ruling out the possibility of a strong causal relationship. In summary, it is not yet clear how imbalances in motor pathway excitability or even noticeable muscle contractions are related to the illusion and adaptation effects. Gilhodes and colleagues suggested that illusory phenomena might *cause* some muscle activity, rather than be its consequence. Kito and colleagues emphasize the activity in motor pathways without clearly explaining its role in perception.

To conclude our discussion of models of adaptation in proprioceptive channels, it is worth noting that only *two* opponent channels encoding direction of movement would suffice to explain the findings in the current study because movements in the elbow joint are limited to two directions. For joints that allow multidirectional movements e.g. wrist, a distribution-shift model would be more appropriate. According to the distribution-shift model of motion in vision [22], all the channels are involved in perception of direction, rather than only the channels coding two opposite directions.

### Potential limitations of vibration as a stimulus

Even if we ignore the fact that vibration does not activate all the afferents which can contribute to movement perception [30], [31], there is a profound difference between neural signals induced by vibration and those occurring during natural passive movement. Continuous vibration of the elbow flexor muscles signifies a prolonged period in which the arm is continuously extending and this is not anatomically possible. A unique aspect of our results, the long periods of absence of perceived movement, could partly be due to this property of the stimulus. On the other hand, the similarity between adaptation to muscle vibration and adaptation to visual motion seems even more remarkable if the difference between the respective stimuli is taken into account. Visual stimuli mimic the situations that occur in the natural environment and early descriptions of the motion aftereffect were in fact based on natural events, such as streaming of water or a parade [32].

### Conclusion

We report perceptual consequences of prolonged stimulation of a proprioceptive movement channel, and place them in a theoretical framework that attributes functional significance to them. The changes we observed can be summarized as a reduced ability to perceive movement and the shift from a clear perception of one-directional movement towards a multistable perceptual state in which extension alternates with flexion and no-movement periods during invariant stimulation. The aftereffect of stimulation, in absence of proprioceptive afferent activity, restores a perception of movement in the direction opposite to that perceived during stimulation. We propose that these changes are parts of an adaptive process which functions to keep the organism in tune with the environment and to best use the information capacity of the sensory system.

Two broad directions seem to be promising in this little explored line of research. One is the exploration of mechanisms common to different modalities–vision, proprioception and possibly other-in processing of dynamic stimuli. The other is to explore issues specific to propriception, such as the relationship between sensory *and motor* channels on one side, and adaptation and related phenomena in conscious perception on the other.

## Methods

### Participants

Twelve participants completed the main experiment, including two authors (JT and JS). They were volunteers recruited from staff at the Prince of Wales Medical Research Institute and psychology undergraduates at the University of Sydney. Seven participants completed a follow-up experiment, including three authors (TSC, JT and JS). The study was approved by the Human Research Ethics Committees of the University of New South Wales and University of Sydney and all the participants (except for the authors) signed the informed consent.

### Apparatus and procedure

A custom-built wooden board supported the left forearm in the horizontal plane, approximately 10 cm below the shoulder level, at 130° relative to the upper arm. The 90-Hz vibrator (Breville HM500) was attached to the side of the board, and its head held against the biceps tendon using an elastic band.

The vibration was applied for 6 minutes (**Vibration**), followed by 2 minutes of post-vibration period (**Post-vibration**). Participants closed their eyes and used the right hand to signal movement about the left elbow with two keys on a standard keyboard. The index finger indicated movement to the left, and the middle finger movement to the right. The rate of presses indicated the relative speed of perceived movement. The Vibration-Post-vibration cycle was performed twice, with at least 3 min break between the runs. Before experimental runs, participants received a short practice in which they varied the frequency of presses from slow to fast. In a control condition performed at the end of the session, they were asked to press one key at a constant rate for 6 minutes.

### Instructions

Participants were informed about the illusory nature of the perceived movement evoked by vibration. They were to try to keep their arm relaxed and be open to any of the following possibilities: the illusion of movement may not occur; if it occurs, it may, over time, change direction or speed, and finally the illusion may cease altogether and reappear. They were asked to press a key to indicate movement to the left or right only if they felt the movement clearly, and to stop responding if in doubt. They were told that sensations during and after vibration were equally important to signal. Finally, they were advised that their perception of arm movement may not be accompanied by a perception of displacement and that they should focus on movement only.

Follow-up study. With subjects in the same set-up and receiving the same instructions as described above, vibration was applied to the biceps tendon for 3 min with a post-vibration period of 2 min. In this study, EMG was recorded from biceps and triceps via self-adhesive surface electrodes fixed over the muscle bellies. EMG was amplified, filtered (16–1000 Hz; CED 1902 amplifiers) and sampled to computer (2000 Hz) via a laboratory interface (CED 1401, Spike2 software, Cambridge Electronic Design).

### Data analysis

Raw data were the number of keypresses per second. These indicated the perceived relative speed of arm movement, with one key indicating elbow **extension**, and the other, **flexion**. We derived three measures from running averages based on 3-s time periods: A. Probability of responding as a function of time, or the number of participants that had pressed one or other key at a given time. The probability was measured with one-second precision, using running averages as described above. B. Standardized response rate indicating perceived velocity. Data were normalized to account for individual differences in the absolute number of responses per second. For each participant, all the responses in both runs were normalized to the greatest response frequency (assigned a value of 1) indicating elbow extension during the vibration period of Run 1. From this we computed a) overall perceived velocity, using sign to indicate direction of movement (positive for extension and negative for flexion) and zero to indicate periods in which no movement was perceived; b) perceived velocity during movement, calculated separately for extension and flexion responses, and excluding periods of no movement. C. Duration of periods when uninterrupted movement (extension or flexion) was perceived, or when no movement was perceived. A 3-s period during which neither key was pressed counted as an interruption.

For the follow-up study, root mean square (rms) EMG was calculated for each 1-s interval during vibration and post-vibration. These measures were smoothed using a 5 point running average before correlation with the perceived velocity of movement for each subject.
